# Effect of Salicylic Acid and Structurally Related Compounds in the Accumulation of Phytoalexins in Cotyledons of Common Bean (*Phaseolus vulgaris* L.) Cultivars

**DOI:** 10.3390/molecules180910609

**Published:** 2013-09-02

**Authors:** Diego Durango, Natalia Pulgarin, Fernando Echeverri, Gustavo Escobar, Winston Quiñones

**Affiliations:** 1 Grupo de Química de los Productos Naturales y los Alimentos, Escuela de Química, Facultad de Ciencias, Universidad Nacional de Colombia-Sede Medellín, Calle 59ª 63-020 Autopista Norte, P.O. Box 3840, Medellín, Colombia; 2 Química Orgánica de Productos Naturales, Facultad de Ciencias Exactas y Naturales, Universidad de Antioquia, Calle 70 N° 52-21, P.O. Box 1226, Medellín, Colombia

**Keywords:** cultivars, elicitor, phaseollin, dose-response profiles, *Colletotrichum lindemuthianum*, antifungal activity, dihydro-quinazolinones

## Abstract

In the present work, isoflavonoid phytoalexin production in response to the application of salicylic acid in cotyledons of four common bean (*Phaseolus vulgaris*) cultivars (SA) was evaluated. The time-course and dose-response profiles of the induction process were established by quantifying the isoflavonoids by HPLC. Cotyledons of anthracnose-resistant cultivars induced by SA produced substantially higher phytoalexin contents as compared to the susceptible ones. In addition, maximum levels of phytoalexins (50–100 fold increases) were reached between 96 and 144 h, and when a concentration of SA from 3.62 to 14.50 mM was used. The observations also indicate that there was a relatively good correlation between the phytoalexin contents and the inhibitory effect against *C. lindemuthianum*; the higher antifungal activity was observed during the first 48 hours for extracts from cotyledons treated with SA at 1.45 and 3.62 mM, and between 96 and 144 h after induction. Finally, compounds structurally related to SA (dihydro-quinazolinones and some imines) showed a strong elicitor effect. Moreover, induced extracts from cotyledons treated with these potential elicitors, besides the properly elicitors, displayed a weak to moderated antifungal activity. These compounds may be considered good candidates for developing of new phytoprotectants. Furthermore, phytoalexin-eliciting substances may contribute for selecting disease resistant cultivars.

## 1. Introduction

The common bean (*Phaseolus vulgaris* L.) is the most important edible food legume in the World; it represents 50% of the grain legumes consumed worldwide and provides 15% of the protein and 30% of the caloric requirement to the World population [1]. The common bean also represents a valuable source of vitamins, minerals and fiber, especially for the poorer people of Africa and Latin America [2]. Unfortunately, this crop is seriously affected by several fungal pathogens that can cause severe yield losses. Among these fungal diseases, the most widespread and destructive, especially in cool weathers in Latin America and Africa, is anthracnose (caused by *Colletotrichum lindemuthianum*, Sacc. & Magnus, Scribner) that affects yield, seed quality, and marketability of beans. 

Traditionally, anthracnose has been successfully controlled through the application of synthetic fungicides, but there is a growing global concern over the continuous use of non-selective synthetic chemicals on food crops because of their potential deleterious effects on human health and the environment. Also, the development of fungicide resistance by pathogens may increase production costs resulting of the need for apply higher more frequent doses to the crops, and subsequently causing environmental problems due to the presence of fungicide residues in foods [3]. In an attempt to modify this situation, researchers are actively working in alternative methods of control of fungal diseases. Within this context is the utilization of elicitors, compounds that are able to stimulate the natural defense mechanisms in plants, including the biosynthesis and accumulation of fungitoxic compounds (named phytoalexins) [4,5]. Due to their non-biocidal character and selective action, elicitors offer ecological advantages over synthetic fungicides [6,7]. Moreover, elicitors can serve as versatile tools for selecting disease resistant cultivars and provide valuable information about the plant-pathogen relationship. 

Salicylic acid (SA), an endogenous elicitor, plays a crucial role in plant growth and development, and in the induction processes of systemic acquired resistance (SAR) [8,9]. SA is involved in signal transduction systems, which stimulate particular enzymes catalyzing biosynthetic reactions to produce defense compounds [10], which thus may provide protection for plants against pathogens. Exogenous application of SA can also result in the induction of defense compounds and consequently resistance against pathogens [11,12]. Furthermore some derivatives and analogues of SA, such as 2,6-dichloroisonicotinic acid (INA) and acibenzolar-*S*-methyl (BTH), resemble SA by acting as exogenous chemical inducers of SAR and protecting plants from infections by fungal, bacterial, or viral pathogens [13,14,15,16,17]. 

**Figure 1:**
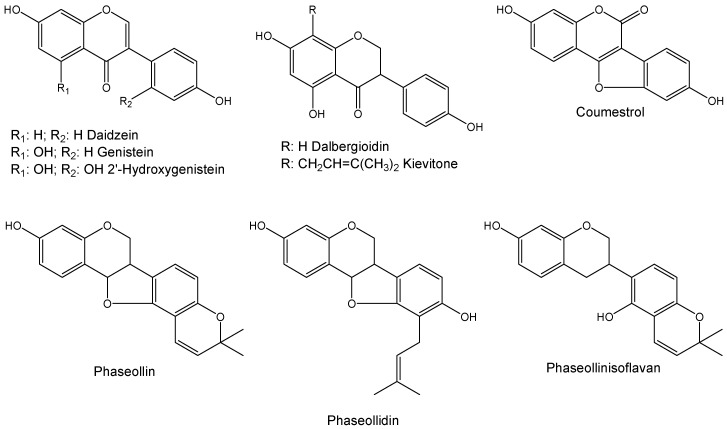
Chemical structures of some phytoalexins in common bean.

Previous works have showed that the application of some elicitors, including CuCl_2_ [18], chitosan [18,19], arachidonic and linoleic acid [20], accessions of *Pseudomonas syringae* pv. *phaseolicola* [21], rhizobacteria [21], *Fusarium solani* f. sp. *phaseoli* [21] and plant hormones [22] on *Phaseolus vulgaris* induce the isoflavonoid phytoalexin accumulation like genistein, daidzein, 2-hydroxygenistein, dalbergioidin, phaseollin, phaseollidin, phaseollinisoflavan, kievitone and coumestrol (Figure 1). The aims of this study were to investigate the phytoalexin accumulation and inhibitory effects against *C. lindemuthianum* of extracts from common bean cotyledons of four cultivars with different phytopathological behavior towards anthracnose, treated or not with SA. Additionally, the effect in the phytoalexin accumulation on cotyledons of common bean using some structurally related compounds to SA, like dihydroquinazolinones and some imines was evaluated.

## 2. Results and Discussion

### 2.1. Time-Course Phytoalexin Accumulation

Elicitors have been recognized as an attractive alternative to non-selective fungicides in crop protection. Exogenous application of elicitors can induce localized or systemic resistance in susceptible plants, which become resistant to later infections [6,8]. In addition, elicitors can be used alone or in combination with fungicides, reducing the amounts necessary to control pathogenic microorganisms and the undesirable environmental side effects associated with the indiscriminate use of fungicides. Besides their potential use for crop protection, elicitors can serve as versatile tools for selecting disease resistant cultivars. 

**Figure 2:**
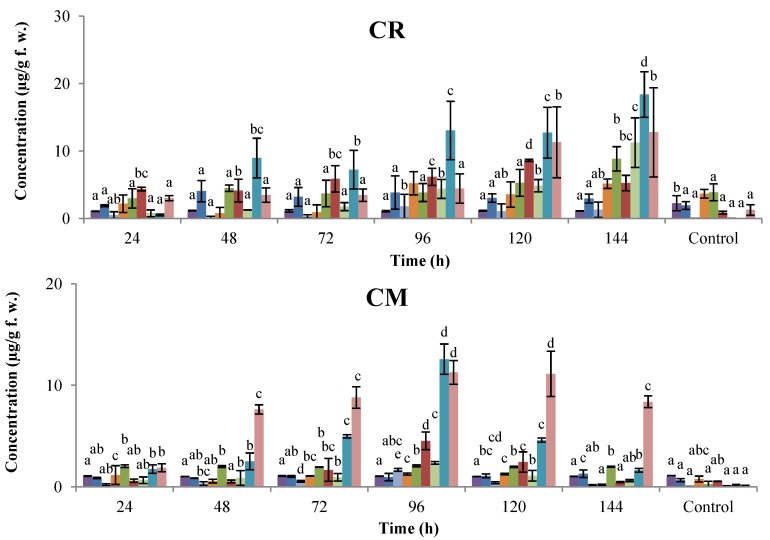

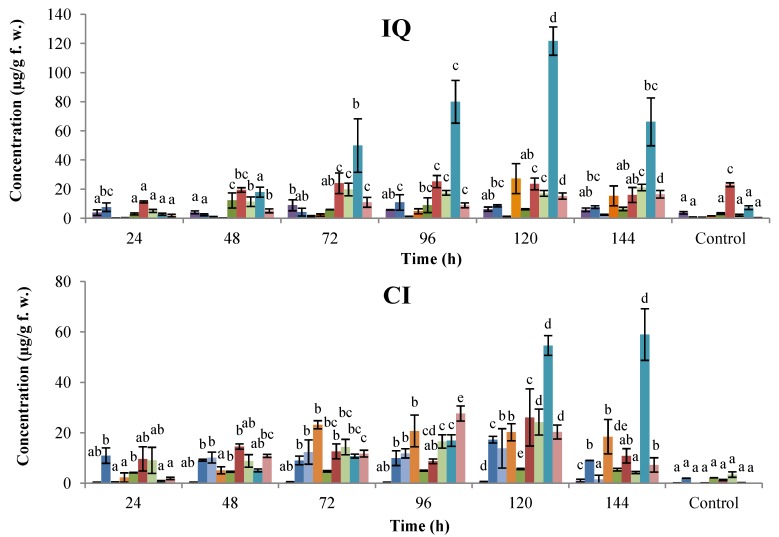
Time-course accumulation of phytoalexins in common bean cotyledons treated with SA. 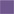, genistein; 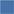, dalbergioidin; 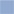, phaseollinisoflavan; 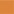, phaseollidin; 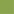, daidzein; 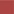, 2’-hydroxygenistein; 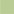, kievitone; 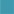, coumestrol; 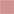, phaseollin. Bars represent the mean concentrations of the isoflavonoids ± standard deviation (n = 3). Cultivars: CM, Cargamanto Mocho; CR, Cargamanto Rojo; IQ, ICA Quimbaya; CI, CORPOICA 106. For each compound, bars with different letters are significantly different (*p* = 0.05; Fisher’s LSD test).

To investigate the effect of application of SA in the course-time, cotyledons of four common bean cultivars (two anthracnose-resistant: CORPOICA 106 and ICA Quimbaya, and two antracnose-susceptible: Cargamanto Mocho and Cargamanto Rojo) were elicited with 1.45 mM SA during 4 h. Then, cotyledons were extracted every 24 h for six consecutive days and the corresponding extracts analyzed by HPLC. Concentration of phytoalexins in the course time is shown in Figure 2. As can be seen, exogenous elicitation of cotyledons with SA resulted in a dramatic increase in the phytoalexin levels, mainly coumestrol, phaseollin, 2'-hydroxygenistein, and kievitone, in relation to the controls. Analysis indicates that the phytoalexin content varied significantly (*p* = 0.05) between treatments, except for dalbergioidin, genistein, and phaseollinisoflavan in anthracnose-susceptible cultivars. In general, phytoalexin accumulation increased progressively according to the post-induction time; the highest amounts of phytoalexins were accumulated between 96 and 144 h. Otherwise, contents of genistein and daidzein, precursors of phaseollin and kievitone respectively, along with dalbergioidin remained almost constant over the whole period of the evaluation (144 h). Results show that coumestrol was the major metabolite elicited in all varieties. Maximum concentrations of this coumestan in anthracnose-susceptible cultivars were about 18.40 and 12.57 µg/g for Cargamanto Rojo and Cargamanto Mocho, respectively. Meanwhile for ICA Quimbaya and CORPOICA 106, coumestrol reached in that order, levels of 121.67 and 58.94 µg/g. On the other hand, kievitone presented maximum values of 11.26 and 2.36 µg/g for Cargamanto Rojo and Cargamanto Mocho, whereas cotyledons from resistant cultivars accumulated amounts of 21.20 (for ICA Quimbaya) and 29.27 µg/g (for CORPOICA 106). Overall, the amount of phytoalexins in resistant cultivars was higher than that found in cotyledons of susceptible ones. 

### 2.2. Dose–Response of Elicitor Treatments

**Figure 3:**
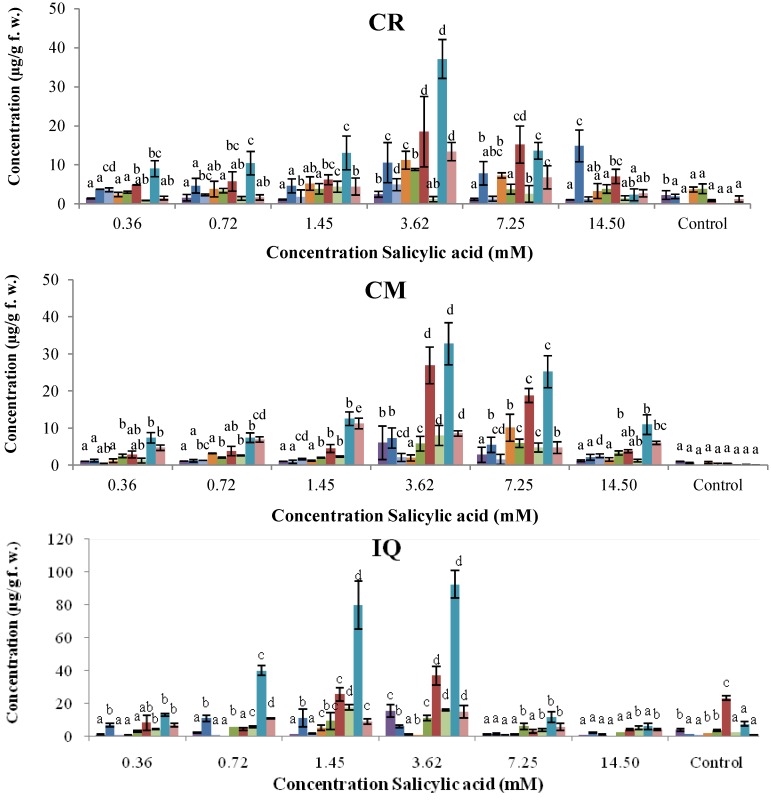

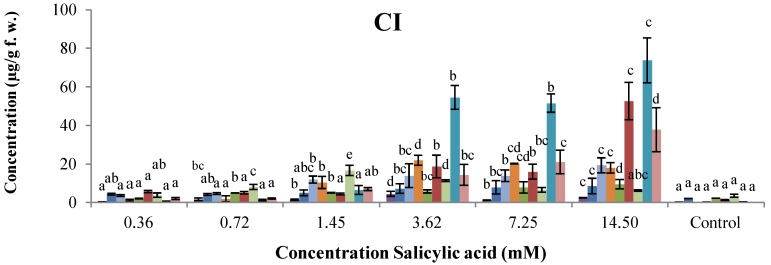
Accumulation of isoflavonoid phytoalexins on cotyledons of *Phaseolus vulgaris* by SA at different concentrations. 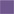, genistein; 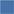, dalbergioidin; 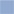, phaseollinisoflavan; 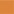, phaseollidin; 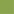, daidzein; 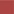, 2’-hydroxygenistein; 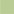, kievitone; 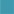, coumestrol; 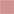, phaseollin. Bars represent the mean concentrations of the isoflavonoids ± standard deviation (n = 3). Cultivars: CM, Cargamanto Mocho; CR, Cargamanto Rojo; IQ, ICA Quimbaya; CI, CORPOICA 106. For each compound, bars with different letters are significantly different (*p* = 0.05; Fisher’s LSD test).

The use of SA as a plant defense inducer is limited by its phytotoxic activity. The effect of the concentration of SA on the synthesis of phytoalexins on common bean cotyledons was evaluated in the 0.36–14.50 mM range and after 96 h of incubation (Figure 3). All cultivars showed significant increases in the phytoalexin levels after treatment with SA in relation to the control. Even at 0.36 mM SA, amounts of coumestrol were between 2- and 6-fold above that in the corresponding controls. In general, levels of phytoalexins in cotyledons increased steadily at 3.62 mM SA and below, in a dose-dependent manner. As shown in Figure 3, phytoalexin contents progressively increased to reach their maximum concentrations at 3.62 mM SA for Cargamanto Rojo, Cargamanto Mocho, and ICA Quimbaya. Then at 7.25 mM SA, phytoalexin concentrations declined slightly for Cargamanto Mocho, and rapidly for Cargamanto Rojo and ICA Quimbaya. Unlike these cultivars, CORPOICA 106 reached the maximum level of phytoalexins at 14.50 mM SA. However after 96 h and at concentrations of 3.62 mM SA and above, common bean cotyledons started to show symptoms of necrosis, whereas controls and treatments below 3.62 mM SA remained green for this period (data not shown). Most of cotyledons treated with 7.25 mM SA turned brownish and exhibited wilting. Accordingly, under these conditions a hypersensitive response occurred, and our results suggest that SA at 3.62 mM and below, is safe and could be used as elicitor in common bean. Nonetheless, further studies about the physiological effects of SA in common bean cotyledons (and other tissues) are needed. 

On the other hand, cotyledons from resistant cultivars (ICA Quimbaya and CORPOICA 106) accumulated significantly higher amount of phytoalexins as compared to the susceptible cultivars (Cargamanto Mocho and Rojo). For example, coumestrol reached maximal levels of about 37.11 and 32.75 µg/g for Cargamanto Rojo and Cargamanto Mocho, and near 73.65 and 92.37 µg/g for CORPOICA 106 and ICA Quimbaya respectively; almost two- and three times higher phytoalexin production in the resistant cultivars in comparison with the susceptible ones. It is noteworthy that cotyledons of CORPOICA 106 accumulated high amounts of phaseollidin and phaseollinisoflavan, being respectively about 13 and 20-fold higher that in the other cultivars. However, no substantial difference was observed in the amount of daidzein and genistein between resistant and susceptible cultivars. 

SA is a recognized natural elicitor involved in the disease resistance of plants. It may improve the plant defensive capacity against a broad array of pathogens after appropriate treatment. In this study, the application of SA on bean cotyledons resulted in strong increases of phytoalexin content and antifungal activity of the extracts in comparison to controls. The data indicate that SA activated phytoalexin biosynthesis in all cultivars. Coumestrol, phaseollin, and 2'-hydroxygenistein were the major phytoalexins induced in cotyledons from Colombian bean varieties. This contrasts with an earlier study, in which kievitone was the major phytoalexin in cotyledons, whereas phaseollin predominates in hypocotyls [18]. In addition, it was found that anthracnose-resistant cultivars treated with SA accumulated significantly higher levels of phytoalexins as compared to the susceptible ones. These results are in accordance with other results reported for common bean elicited by CuCl_2_ [18] and some other crops [23,24,25,26]. Hence, the induction of bean cotyledons with SA may contribute to the development of rapid and low-cost techniques for selecting resistant cultivars to anthracnose based on the biosynthesis of phytoalexins. According to course-time experiments, as a result of induction with SA, there was a gradual increase in phytoalexin content mainly for coumestrol, phaseollin, kievitone, 2'-hydroxygenistein, phaseollidin and phaseollinisoflavan. Meanwhile, genistein, daidzein, and dalbergioidin contents, precursors of phytoalexins kievitone, coumestrol and pterocarpans, remained almost constant over the whole period of the evaluation. Otherwise, dose-response experiments showed that when cotyledons were immersed on SA at 3.62 mM and below, the phytoalexin production was increased in a dose-dependent manner; this was appreciably higher for anthracnose-resistant cultivars. However, the application of SA at high concentrations (7.25 and 14.50 mM) resulted in a fast decline of phytoalexin contents, except for the cultivar CORPOICA 106. We hypothesize that the decreases in the biosynthesis of defense secondary metabolites can result in some effects related with the phytotoxic character of SA, which has been well documented [27,28]. The above results are in agreement with the necrosis symptoms observed in cotyledons treated with SA at 7.25 and 14.50 mM. Nevertheless, the molecular mechanism by which SA at high concentration inhibited the phytoalexin formation is unclear. Previously, War *et al.* [29] found that chickpea plants treated with SA at 2 mM showed phytotoxicity symptoms and a lower peroxidase and polyphenol oxidase activity, and phenol content. Accordingly, they suggested that SA at 1.5 mM is safe to these plants and could be utilized as plant defense inducer. 

Thus, it was observed that the highest levels of phytoalexins in common bean cotyledons were achieved after 96 h of post-induction and using SA at 3.62 mM (for cv. Cargamanto Rojo, Cargamanto Mocho, and ICA Quimbaya) and at 14.50 mM (for CORPOICA 106). Interestingly, the phytoalexin amounts detected in our study from Colombian common bean cultivars are substantially lower compared with those reported for North American and European cultivars. Hynes *et al.* [21] reported accumulations of kievitone reaching 850 ± 251 µg/g f.w in wounded cotyledons (white bean cv. OAC Seaforth) following inoculation with *Fusarium solani* f. sp. *phaseoli*. 

### 2.3. Antifungal Activity

**Figure 4:**
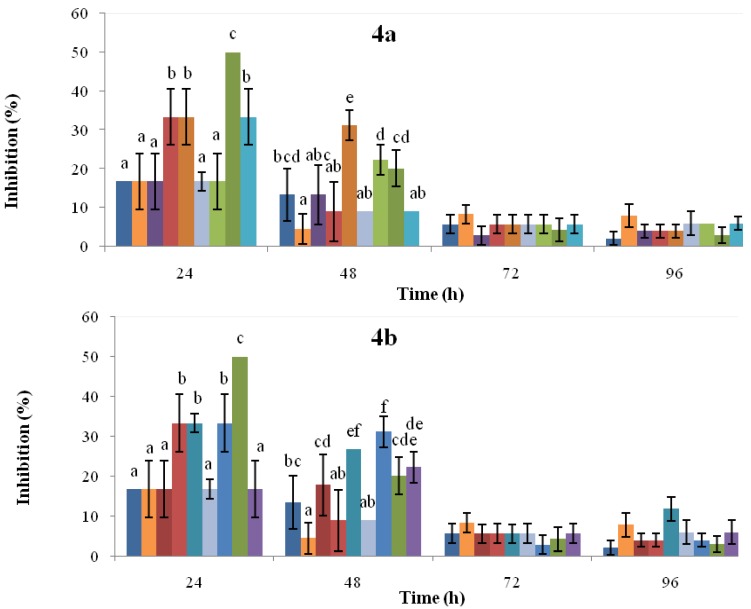
Antifungal activity against *C. lindemuthianum* of extracts from common bean cotyledons induced by SA at different concentration (up) and post-induction time (down). Cultivars: CR, Cargamanto Rojo; IQ, ICA Quimbaya. Figure 4a: 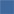, solvent control; 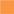, CR-untreated cotyledons; 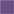, CR-0.72 mM SA; 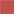, CR-1.45 mM SA; 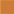, CR-3.62 mM SA; 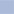, IQ-untreated cotyledons; 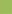, IQ-0.72 mM SA; 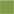, IQ-1.45 mM SA; 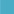, IQ-3.62 mM SA. Figure 4b: 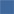, solvent control; 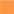, CR-untreated cotyledons; 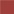, CR-48 h post-induction; 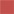, CR-96 h post-induction; 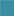, CR-144 h post-induction; 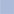, IQ-untreated cotyledons; 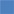, IQ-48 h post-induction; 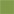, IQ-96 h post-induction; 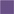, IQ-144 h post-induction. For each time point, the bars headed by the same letter do not differ at *p* = 0.05 (Fisher’s LSD test).

The antifungal activity of extracts obtained from cotyledons (course-time experiments at 48, 96, and 144 h; dose-response assays at 0.72, 1.45, y 3.62 mM) of common bean cultivars Cargamanto Rojo (susceptible to anthracnose) and ICA Quimbaya (resistant to anthracnose), non-treated and treated with SA, in terms of radial growth inhibition of *C. lindemuthianum* are summarized in Figure 4. The inhibition of *C. lindemuthianum* growth was dependent to the concentration of SA used in treatments and the incubation (post-induction) time. In general, the radial growth was inhibited in higher proportion during the first 24 h. As shown in Figure 4a (top), inhibitions of *C. lindemuthianum* using extracts from both cultivars treated with SA at 1.45 and 3.62 mM were significantly higher compared to the untreated cotyledons. At 24 h, it can be noticed that the extract obtained from cotyledons of ICA Quimbaya treated with 1.45 mM SA was more active (inhibition about 50%) toward *C. lindemuthianum* than the extract proceeding from Cargamanto Rojo (inhibition 33%) under the same conditions. Nonetheless, the antifungal activity of cotyledons treated with 3.62 mM SA was similar for both cultivars. On the other hand, cotyledons of both cultivars elicited by 0.72 mM SA showed no substantial differences with respect to untreated cotyledons and solvent control. Thus, the increase in the radial growth inhibition of *C. lindemuthianum* seems to be related with the upper phytoalexin levels presents in the extracts. Even so, the comparable antifungal effect against *C. lindemuthianum* shown by Cargamanto Rojo and ICA Quimbaya cotyledons induced with SA at 3.62 mM results strange given their so different chemical profiles.

From Figure 4b (bottom), it can be seen that extracts from ICA Quimbaya cotyledons induced by 1.45 mM SA and incubated during 48 and 96 h showed radial growth inhibitions of 33.3 and 50.0% respectively. However, extracts obtained after 144 h presented less antifungal activity and no significant differences were found between this extract, untreated cotyledons, and the solvent control. This behavior in the antifungal activity correlates with the increased amounts of isoflavonoid phytolalexins detected in ICA Quimbaya cotyledons 96 h post-induction. Meanwhile, it can be found that extracts from Cargamanto Rojo cotyledons treated with 1.45 mM SA and incubated for 96 and 144 h showed inhibitions of about 33.3%. The above is in agreement with the higher levels of phytoalexins detected for these extracts as compared to untreated cotyledons and that obtained 48 h post-induction. In addition, Figure 4 showed that inhibitory effects of extracts rapidly decreased with time, a fact that suggests that the fungus had a rapid adaptation to the medium. The above is consistent with the known capacity of phytopathogenic fungi, including *C. lindemuthianum*, to circumvent some of the plant chemical defenses through metabolism and detoxification [30]. However, further studies are necessary to establish the relationship between phytoalexin level and the inhibitory effects against *C. lindemuthianum*. 

Our study also indicates that the activity of extracts from bean cotyledons treated with SA against *C. lindemuthianum* was only slightly enhanced. Nonetheless, it is important to note that the extracts were evaluated without an additional purification and at a relatively low concentration (<300 µg/mL), and in a static experimental system that not allow generation of new amounts of phytoalexins. In general, there were no significant differences in the fungitoxicity against *C. lindemuthianum* between untreated cotyledons and those treated at 0.62 mM SA, at least during the first 24 h of evaluation. Likewise, extracts obtained after 48 h (for Cargamanto Rojo) and 144 h (for ICA Quimbaya) of induction showed a similar inhibitory effect than untreated cotyledons (about 16%). The results also demonstrate that the application of 1.45 and 3.62 mM SA on cotyledons or s post-induction time of 96 and 144 h for Cargamanto Rojo and 48 and 96 h for ICA Quimbaya led to more active extracts (inhibitions between 33 and 50%). These findings are in agreement with the greater phytoalexin contents established for cotyledons elicited by 1.45 and 3.62 mM SA and post-incubation times longer than 96 h, in relation to untreated controls. Under these conditions, cotyledons of Cargamanto Rojo and ICA Quimbaya were found to accumulate the higher levels of coumestrol, 2’-hydroxygenistein, phaseollin, and kievitone, among other. Nonetheless, no marked differences in the antifungal activity were found between extracts from anthracnose-resistant and anthracnose-susceptible cultivars. Moreover, experimental data also suggest a rapid adaptation of phytopathogenic microorganisms to the medium containing the different extracts; the inhibitory effect for all extracts against *C. lindemuthianum* was nearly the same after 72 h of evaluation (<10% inhibition). It seems that the use of extracts without further purification and at low concentrations (<300 µg/mL) could be responsible for the quick detoxification of the medium by *C. lindemuthianum*, besides the modest antifungal activity after 72 h. Extracts at these concentrations may contain fungitoxic compounds at very low levels, being rapidly transformed into innocuous metabolites by biotransformation after 72 h. Thus, the lack of antifungal activity of extracts after 72 h may be due to metabolism of the phytoalexins by the fungus. 

### 2.4. Inducer and Antifungal Effects of Structurally Related Compounds to SA

**Figure 5:**
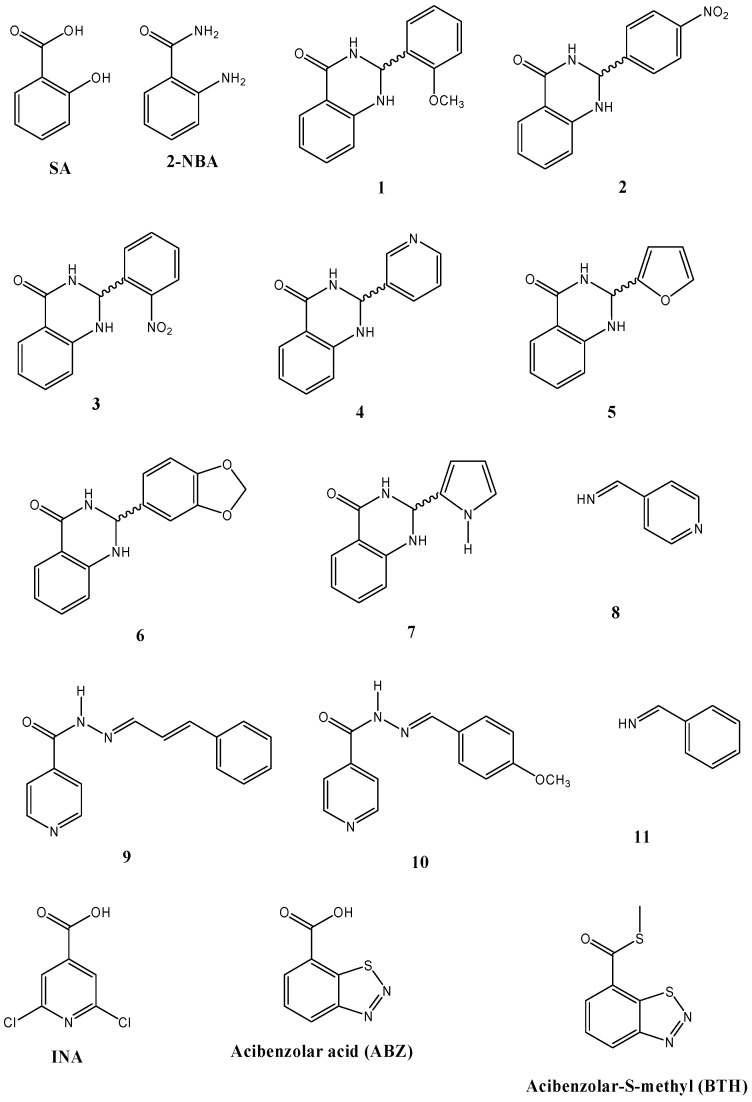
Chemical structures of compounds evaluated as elicitors.

Currently, there is an increasing interest in the search for new elicitors for controlling important plant diseases. Here, the inducer effect of phytoalexins in bean cotyledons of some dihydro-quinazolinones and imines (Figure 5), along with ABZ, INA, BTH, and 2-NBA was evaluated. Bean phytoalexins were grouped in three classes: isoflavones and isoflavanones (genistein, daidzein, dalbergioidin, 2’-hydroxygenistein, and kievitone), coumestan (coumestrol), and pterocarpans and isoflavans (phaseollidin, phaseollin, and phaseollinisoflavan). As can be seen in Table 1, dihydro-quinazolinones and imines possess a strong elicitation effect, being even higher than that shown by the plant hormone, SA, and the structurally related compound, 2-NBA. The upper isoflavones/isoflavanones accumulation was found to be induced by **1**, followed by **10** and **9**. The isoflavones/isoflavanones content detected in response to **1** was near twice that found in cotyledons treated with SA. In addition, **1** induced high levels of coumestrol (73.55 µg/g f.w., the highest amount) and pterocarpans/isoflavan. 

In fact, coumestrol content using **1**, **6** and **10** as elicitors was increased in that order by about six, five, and three-fold in comparison to the cotyledons induced by SA. It is noteworthy that coumestrol was not detected in appreciable amounts in untreated cotyledons. Overall, pterocarpans/isoflavan levels of common bean cotyledons in response to dihydroquinazolinones and imines were always higher than that detected when SA was used as elicitor; only **2**, **3**, **5**, and **6** showed a slightly higher increases in pterocarpans/isoflavan content. 

Meanwhile, **10** had a stronger inducer effect on the pterocarpans/isoflavan content because these increased almost ten and five-fold the levels detected in untreated (control) and SA-treated cotyledons respectively. It is noteworthy that extracts proceeding from common bean cotyledons cv. Cargamanto Mocho treated with **10** also showed the higher inhibitory effects against *C. lindemuthianum* during the first 48 h. 

Furthermore, although bean cotyledons treated with **6** resulted in a marked increase of coumestrol, the antifungal activity was relatively weak (55.9 ± 8.8 and 44.2 ± 20.9 after 24 and 48 h, respectively). This result suggests a low fungitoxic effect of coumestrol against *C. lindemuthianum*. In general, inhibitory effect of extracts was rapidly decreased; at 24 h, fungal inhibitions were almost twice that found after 48 h except for **6** and **7**.

Additionally, we also evaluated the direct antifungal properties of these potential elicitors. In general, dihydroquinazolinones and imines displayed a moderate to weak fungistatic activity against *C. lindemuthianum*. As can be seen in Table 1, the highest radial growth inhibition was exhibited by **10** (76.9% after 48 h at 200 μg/mL), followed by **8** and **6**. Remarkably, **10**, **6**, and **1**, which showed higher phytoalexin-inducing effect, also displayed inhibitions of 76.9 ± 0.0, 57.7 ± 13.3, and 38.5 ± 6.7, so dihydroquinazolinones and imines may have dual mode of action for controlling of fungal diseases; elevating host resistance and reducing pathogen inoculum. 

**Table 1:** Elicitor effect of some dihydro-quinazolinones and imines related structurally to SA (at 1.45 mM and after 96 h post-induction) in cotyledons of common bean cv. Cargamanto Mocho, and antifungal activity of the extracts from induced cotyledons.

Compound	Isoflavones/Isoflavanones * (µg/g f.w.)	Coumestan (µg/g f.w.)	Pterocarpans/Isoflavan † (µg/g f.w.)	Radial growth inhibition (%) of *C. lindemuthianum*		
				Elicited-cotyledon extracts		Elicitor
				24 h	48 h	48 h
**SA**	20.20 ± 1.47 ^d^	13.07 ± 1.49 ^a^	11.53 ± 1.42	n.d.		9.5 ± 4.1
**2-NBA**	22.97 ± 2.33 ^a,c,d^	13.72 ± 1.12 ^a^	17.72 ± 5.45	n.d.		n.d.
**1** ^§^	43.04 ± 9.36 ^a,b,c,d^	79.99 ± 22.02 ^a,b,c,d^	46.67 ± 10.52 ^a,b,c,d^	82.4 ± 0.0	33.7 ± 19.8	38.5 ± 6.7
**2** ^§^	15.57 ± 3.23	12.93 ± 6.68 ^a^	13.73 ± 2.85	38.2 ± 8.8	19.8 ± 3.5	11.5 ± 6.7
**3** ^§^	20.16 ± 4.06 ^b,c^	14.37 ± 0.20 ^a^	19.92 ± 9.89 ^a,c^	38.2 ± 8.8	19.8 ± 3.5	42.3 ± 0.0
**4** ^§^	24.13 ± 6.84 ^a,c,d^	14.98 ± 1.75 ^a^	34.90 ± 16.37 ^a,c,d^	47.1 ± 0.0	23.3 ± 0.0	53.8 ± 11.5
**5** ^§^	20.81 ± 6.81 ^c^	21.25 ± 9.26 ^a^	11.94 ± 5.21	52.9 ± 5.1	26.7 ± 10.5	42.3 ± 0.0
**6** ^§^	29.26 ± 14.19 ^a,b,c,d^	68.58 ± 18.89 ^a,b,c,d^	12.05 ± 8.51	55.9 ± 8.8	44.2 ± 20.9	57.7 ± 13.3
**7** ^§^	17.64 ± 6.46 ^c^	24.81 ± 9.79 ^a^	36.03 ± 9.33 ^a,c,d^	64.7 ± 0.0	58.1 ± 7.0	38.5 ± 6.7
**8** ^‡^	17.96 ± 2.86 ^a,b,c^	23.08 ± 3.11 ^a^	28.29 ± 5.00 ^a,c,d^	64.7 ± 0.0	33.7 ± 3.5	65.4 ± 0.0
**9** ^ǂ^	32.60 ± 16.75 ^a,b,c,d^	18.02 ± 2.69 ^a^	26.96 ± 4.08 ^a,c,d^	47.1 ± 0.0	23.3 ± 0.0	50.0 ± 0.0
**10** ^ǂ^	37.88 ± 11.98 ^a,b,c,d^	38.47 ± 4.40 ^a,b,c,d^	60.85 ± 17.05 ^a,b,c,d^	70.6 ± 10.2	45.3 ± 17.4	76.9 ± 0.0
**11** ^‡^	13.85 ± 3.88	10.99 ± 3.01 ^a^	34.71 ± 6.11 ^a,c,d^	47.1 ± 17.6	33.7 ± 3.5	53.8 ± 0.0
**ABZ**	10.58 ± 1.58	17.74 ± 3.27 ^a,b^	9.32 ± 0.41	n.d.		n.d.
**BHT**	7.90 ± 0.64	7.58 ± 1.83 ^a^	6.19 ± 1.57 ^b^	n.d.		n.d.
**INA**	14.64 ± 2.53	18.58 ± 5.14 ^a^	22.61 ± 6.51 ^a^	n.d.		n.d.
**Control**	9.01 ± 0.52	Traces	5.02 ± 0.33 ^b^	n.d.		n.d.

Data correspond to the mean concentrations of the grouped isoflavonoids ± standard desviation (n = 3). * Dalbergioidin, 2'-hydroxygenistein, daidzein, genistein, kievitone. † Phaseollidin, phaseollinisoflavan, phaseollin. n.d. not determined. ^§^ Dihydro-quinazolinones; ^‡^imines; ^ǂ^ acyl hydrazones. n.d.: not determined. Significant difference (*p* = 0.05) between treatment and control (^a^), INA (^b^), BHT (^c^), and ABZ (^d^).

The results concerning the ability to stimulate the phytoalexin biosynthesis of compounds structurally related to SA showed that dihydroquinazolinones and imines exhibit a strong elicitor effect. Lately, new synthetic compounds have been assayed as elicitors in different plant tissues [31,32]. Thus, some chemicals such as 2,6-dichloroisonicotinic acid (INA) and S-methyl benzo-1,2,3-thiadiazole-7-carbothioic acid (acibenzolar-S-methyl or BTH) have been found to be effective inducers of plant defenses [13,14,15,16,17]. Both compounds were discovered as a result of screening assays of elicitors of broad-spectrum resistance in cucumber (*Cucumis sativus* L.) [15,33]. However, only BTH is commercially available (from Syngenta) under the names Actigard and Bion. The present study clearly indicated that 2-NBA, **2**, **3**, **5** induced phytoalexin levels almost similar to that detected when bean cotyledons were treated with SA, a well-known elicitor; particularly, pterocarpans/isoflavan contents were nearly twice that found in untreated controls. Remarkably, phytoalexin accumulation in response to some dihydroquinazolinones and imines (for example, **1**, **4**, **6**, and **10**) was even higher than that found for SA and some recognized synthetic elicitors, such as INA, ABZ, and BHT. In fact, pterocarpans/isoflavan levels on cotyledons were increased by as much as 106 and 169% by **1** and **10** respectively, compared with INA. Additionally, the amounts of coumestrol and pterocarpans/isoflavan for cotyledons treated with dihydroquinazolinones and imines were higher than for SA. The presence of nitrogen-containing functional groups (such as an aryl amide and a nitrogenated substituent in position 2 resembling the structure of SA) in 2-NBA and dihydro-quinazolinones may be an important structural feature for their phytoalexin-inducing activity. Some of the potential synthetic elicitors that have recently been reported are nitrogen-rich compounds. For instance, synthetic pyrazine-2-carboxamide derivatives were found to act as potential elicitors in tissue cultures of *Ononis arvensis*, *Silybum marianum*, and *Genista tinctoria* [34,35]. Thus, the application of 5-*tert*-butyl-6-chloro-*N*-(3-iodo-4-methylphenyl)pyrazine-2-carboxamide in callus culture of *Genista tinctoria* enhanced the genistin production about 57 times compared to untreated control [35]. Furthermore, the application of 1 µM 2-(2-fluoro-6-nitrobenzylsulfanyl) pyridine-4-carbothioamide significantly increased the production of the isoflavonoids genistin (11.60 mg/g dry weight), daidzein (8.31 mg/g dry weight), and genistein (1.50 mg/g dry weight) on *Trifolium pretense* L. suspension culture after 48 h of application as compared to the control by 152, 151 and 400% respectively, which was recently reported by Kašparová *et al.* [36]. Similarly, 3,5-dichloroanthranilic acid (DCA), efficiently induced defense reactions in Arabidopsis (*Arabidopsis thaliana*) plants to the phytopathogens *Hyaloperonospora parasitica* and *Pseudomonas syringae* [37]. Authors also indicate that the removal of the amino group from DCA significantly reduces its biological activity. Moreover, the synthetic substances 2-pyrazinecarboxylic acid, picolinic acid, 2,6-pyridinedicarboxylic acid, 2,3-pyridinedicarboxylic acid, pyrrole-2-carboxylic acid, oxonic acid, among others, increase the phytoalexin(phytocassanes and momilactone A) contents in rice plants [38]. 

Although SA, 2-NBA, AIN, ABZ and BTH are functionally different compounds, they share some common structural features. Thus, all compounds have the benzoyl fragment, and an adjacent electronegative group (substituted at the 2-position, except for INA). Also, all compounds present an electron withdrawing group bonded to the carbonyl group (forming a carboxylic acid for SA, ABZ and INA, an amide for 2-NBA, and a thioester for BTH). Nevertheless, while SA is an acid compound and 2-NBA is basic, both induce similar phytoalexin contents. Hence, the above results suggest that the acidity/basicity of compounds has no effect on the phytoalexin-inducing character.

The cotyledons exposed to dihydroquinazolinones **2** and **3** produced similar phytoalexin levels than those found when 2-NBA (the synthetic precursor) was used. It indicates that the presence of the *N*-nitrophenyl group in these compounds had no effect on the phytoalexin accumulation. In contrast, dihydroquinazolinones having a *N*-methoxyphenyl (**1**) and *N*-benzodioxoyl (**6**) group improved the synthesis of coumestrol as compared to 2-NBA. Also, **1** elicited in bean cotyledons high contents of pterocarpans/isoflavan. These results suggest that the eliciting effects can be related with the functional features presents in the dihydroquinazolinones. The presence of electron-donating groups (like methoxy-, and methylidendioxy- group) in the *C*-phenyl moiety of the tested dihydroquinazolinones seems to be an important requirement for the coumestrol-eliciting effect. Furthermore, it was observed that the pterocarpans/isoflavan accumulated in a greater concentration when cotyledons were treated with **4** and **7**. Both compounds have a nitrogen-containing heterocyclic ring, pyridine and pyrrole respectively. Interestingly, when the dihydroquinazolinones presented a furan ring instead a nitrogen-containing heterocyclic system (**5** *vs.* **7**) there was a substantial loss of eliciting activity of pterocarpans/isoflavan. On the other hand, acyl hydrazones **9** and **10** had a strong effect on isoflavones/isoflavanones content. Also, **10** enhanced the production of coumestrol and pterocarpans/isoflavan.

In addition, dihydroquinazolinones and imines displayed a moderate to weak direct antifungal activity. Besides their promissing phytoalexin-inducing activity, dihydroquinazolinones and imines **1**, **6**, and **10** also inhibit the radial growth of *C. lindemuthianum* between 38.5 and 76.9%. These results are in agreement with previous reports that establish that Schiff bases and dihydroquinazolinones have antifungal properties [39,40]. Thereby, the direct fungistatic properties of dihydroquinazolinones and imines offer an additional advantage over SA, and other elicitors. Therefore, these compounds have the potential to offer a dual mode of action with both direct inhibitory effects again *C. lindemuthianum* and the capacity of enhance the phytoalexin content, and consequently the plant resistance. These results indicate that the dihydroquinazolinones and imines are promissing elicitors of phytoalexins in common bean cotyledons. To the best of our knowledge, this is the first report about the phytoalexin-inducer effect of dihydroquinazolinones and some imines. 

## 3. Experimental

### 3.1. Reagents

Genistein and daidzein were purchased from Sigma (St. Louis, MO, USA). Dalbergioidin, 2’-hydroxygenistein, coumestrol, phaseollidin, phaseollin isoflavan, and phaseollin were obtained during previous work and identified as described in elsewhere [18]. Salicylic acid (SA) was acquired from Merck (Darmstadt, Germany), while 2-aminobenzamide (2-NBA), benzoic hydrazide and isonicotinic hydrazide were from Sigma-Aldrich Co. Dihydroquinazolinones **1** to **7** were prepared by a conventional reaction between 2-aminobenzamide and aldehydes, followed by further heating with an acid catalyst (AcOH). Imine **11** was obtained from benzoic hydrazide whereas imines **8** to **10** were from produced from isonicotinic hydrazide. Synthetic SA analogs such as 2,6-dichloroisonicotinic acid (INA), benzo-1,2,3-thiadiazole-7-carbothioic acid (ABZ), and the commercially available elicitor benzo-1,2,3-thiadiazole-7-carbothioic acid S-methyl ester (BTH) were also purchased from Sigma-Aldrich Co. 

### 3.2. Plant Material

Anthracnose-susceptible bean cultivars Cargamanto Mocho and Cargamanto Rojo were obtained from Semillas & Semillas Ltda (Medellín, Colombia). Anthracnose-resistant cultivars ICA Quimbaya and CORPOICA 106 were acquired from Semicol Ltda (Bogotá, Colombia) and Corpoica (Corporación Colombiana de Investigación Agropecuaria, La Selva, Antioquia, Colombia). Seeds from each Colombian bean (*Phaseolus vulgaris* L.) cultivar were surface-sterilized for 15 min in NaOCl (2.0%), washed with tap water, and sown in moist vermiculite. Seeds were allowed to germinate for seven days in the darkness and at room conditions. Cotyledons harvested from seedlings were carefully washed with tap water in order to preserve their integrity.

### 3.3. Treatments

#### 3.3.1. Dose-Response Experiments

Cotyledons (10 g) of each bean cultivar were immersed for 4 h in solutions of SA at different concentration (0.36, 0.72, 1.45, 3.62, 7.25, and 14.50 mM). Before preparing all solutions, SA was dissolved in ethanol (0.2%). Bean cotyledons submerged into sterile distilled water were used as controls. Then, cotyledons were placed on moist filter paper in polystyrene trays and covered with stretch film. Materials were stored at room temperature and in the dark during 96 h. Experiments were done at least three times. 

#### 3.3.2. Time-Course Experiments

Cotyledons (10 g) of each bean cultivar were dipped into the solution of 1.45 mM SA for 4 h. Subsequently, plant materials were deposited on moist filter paper in sterile polystyrene box, covered with stretch film and stored at room temperature in the darkness during 24, 48, 72, 96, 120, and 144 h. Bean cotyledons treated with distillated water instead SA solution, and stored during 144 h were used as controls. All assays were carried out at least three times. 

#### 3.3.3. Inducer Effect of Structurally Related Compounds to SA

Some dihydroquinazolinones, obtained from 2-aminobenzamide and aldehydes, and imines (imines and acyl hydrazones) were evaluated for their inducer activity on bean cotyledons. Evaluations were carried out using cotyledons (10 g) of cultivar Cargamanto Mocho, which were dipped into solutions of each imine at 1.45 mM and during 4 h. Solutions were prepared dissolving the dihydro-quinazolinone or Schiff base in 0.2% ethanol. Compounds 2,6-dichloroisonicotinic acid (INA), benzo (1,2,3) thiadiazole-7-carbothioic acid (ABZ), and benzo-1,2,3-thiadiazole-7-carbothioic acid S-methyl ester (BTH) were used as positive controls, whereas sterile distilled water was used as the negative control. Then, cotyledons were deposited on moist filter paper in sterile polystyrene box, covered with stretch film and stored at room temperature in the darkness for 96 h. 

### 3.4. Sample Preparation

Cotyledons were cut and further ground in a mortar with 20 mL of 95% ethanol. Then, the solutions were filtrated through Whatman No. 1 filter paper and centrifuged for 6 min (10,000 rpm). The supernatant solutions were evaporated to dryness under reduced pressure at 40 °C (Rotavapor Buchi R-210 with vacuum controller V-850) and the residue was extracted three times with ethyl acetate (EtOAc, 3 × 20 mL). The organic phases were combined and brought to dryness under reduced pressure. Subsequently, the residue was redissolved in methanol (HPLC-grade MeOH, 5.0 mL), and filtered through a syringe sterile filter with a 0.45-mm pore size (Sartorius Biotech GmbH, Goettingen, Germany). The resulting solution (0.5 mL) was used without further purification for HPLC analysis. The samples were kept in amber glass vials and stored at 4°C until HPLC analysis was carried out. The remaining solutions (4.5 mL) from each replicate were combined, evaporated to dryness at 40 °C under vacuum, and used in antifungal assays.

### 3.5. HPLC Analysis

The phytoalexin analysis was carried out on a Gilson chromatograph equipped with a Gilson model 170 diode array detector, using a Phenomenex Security Guard cartridge C18 (4.0 × 3.0 mm) followed by a Phenomenex Luna 5 μ C18 (2) reverse-phase column (150 mm × 4.6 mm i.d., 5 μm) (Phenomenex, Torrance, CA, USA). The metabolites were eluted at a flow rate of 0.7 mL/min with the solvents A = methanol, and B = 0.5% acetic acid in water, as follows: from 10% A to 70% A in 40 min, then 70% A to 90% A in 20 min, and subsequently by holding for 8 min to reequilibrate the column, for the next injection. Injection volume was 20 µL. Phytoalexins were monitored at the wavelengths of 248, 254, 270, 286 and 310 nm, although diode-array detection was used over a wavelength range of 200 to 500 nm to collect spectral data.

### 3.6. Identification and Quantification of Phytoalexins

Isoflavonoid phytoalexins were characterized by comparing the retention times (Rt) of the authentic samples of dalbergioidin, 2’-hydroxygenistein, daidzein, genistein, coumestrol, and phaseollin with those in the extracts, and by co-elution. Additionally, retention times of these isoflavonoids, along with kievitone, phaseollidin and phaseollin isoflavano were confirmed by liquid chromatography with mass spectrometry detection (LC-MSD) on an HP 1100 series HPLC apparatus (Agilent Technologies, Waldbronn, Germany) interfaced to an HP series 1100 mass selective detector equipped with an API-ES chamber, using positive ion mode, and the same chromatographic conditions as described above. MSD conditions were programmed as follows: capillary voltage, 3 kV; nebulizing pressure, 60 psi; drying gas temperature, 350 °C; drying gas flow, 12 L/min. Retention times of dalbergioidin, 2'-hydroxygenistein, daidzein, genistein, coumestrol, kievitone, phaseollidin, phaseollin isoflavan, and phaseollin were respectively 28.85, 30.50, 31.45, 34.48, 37.50, 40.89, 43.51, 44.15, and 45.78 min. Quantification of phytoalexins was carried out using standard calibration curves (peak areas vs. compound concentration for different concentrations). Five working solutions were prepared for each standard in methanol containing genistein, daidzein, dalbergioidin, 2'-hydroxygenistein, coumestrol, and phaseollin in 1, 10, 25, 50, and 100 mg/L concentrations. For phytoalexins without a pure standard phaseollinisoflavano and phaseollidin, and kievitone, concentrations were respectively estimated from the calibration curves for phaseollin, and dalbergioidin, and adjusted on the basis of differences in molecular weight. Data for each peak were collected using the wavelength that provides a maximum response. The results were expressed as μg phytoalexin/g fresh material and presented as mean values ± standard deviation.

### 3.7. Antifungal Assays

The toxicity of extracts proceeding from SA-treated and untreated cotyledons against *C. lindemuthianum* was evaluated through the poisoned food technique [41]. The fungus was isolated from diseased *P. vulgaris* pods, characterized by morphological analysis, and maintained on potato dextrose agar (PDA) at 4 °C. Resulting extracts from induction experiments (without further purification) obtained as described above were dissolved in dimethylsulfoxide (DMSO, 70 μL) and diluted in Petri dishes (measuring 5 cm in diameter) with PDA (20 mL; concentrations approx. between 100 to 300 μg/mL). Petri dishes with and without DMSO were used as controls (solvent and absolute control, respectively). The Petri dishes were incubated at room temperature and the diameter of mycelial growth was measured each 24 hours. The incubation was stopped when the mycelial mass of control Petri dishes had almost filled it (after 96 h). The relative growth inhibition of the treatments (SA-treated and untreated hypocotyls-roots) compared to the controls was calculated as percentage, using the following formula:

Inhibition (%) = {1 – radial growth of treatment (mm)/radial growth of control (mm)} × 100 (1)

The results are expressed as mean values of three replicates [± standard deviation (SD)]. Additionally, the antifungal activity against *C. lindemuthianum* of potential elicitors (dihydro-quinazolinones and imines) was also evaluated. Assays were performed at 200 μg/mL and under the conditions described above. 

### 3.8. Statistical Analysis

Results were analyzed by a one-way ANOVA and mean values were compared with the Fisher’s least significant differences (LSD) at the 0.05 probability level. 

## 4. Conclusions

It is clear from the above discussions that SA substantially increases the phytoalexin content in common bean cotyledons. As a result of induction, anthracnose-resistant cultivars accumulated higher phytoalexin levels in comparison to anthracnose-susceptible cultivars. Therefore, the analysis of the phytoalexin production in bean cotyledons in response to SA might serve as a tool for selecting disease-resistant cultivars in common bean breeding programs. Our results also suggested that the phytoalexin-inducing effects depend on the concentration, the incubation period, and the chemical nature of the elicitor. Maxima levels of phytoalexins were achieved when SA was applied at 3.62 mM (for Cargamanto Mocho, Cargamanto Rojo, and ICA Quimbaya) and 14.50 mM (for CORPOICA 106), and between 96 to 144 h after elicitation. However, SA at higher concentrations than 3.62 mM caused a rapid decrease of the phytoalexin contents for anthracnose-susceptible cultivars and ICA Quimbaya, and necrosis symptoms were evident on cotyledons. Therefore, SA at 3.62 mM and below, could be safely used as elicitor in common bean. In addition, dihydroquinazolinones and imines were found to possess promissory phytoalexin-eliciting activity; they enhanced the phytoalexin amounts and fungistatic properties on extracts from elicited cotyledons. In addition, dihydroquinazolinones and imines displayed a moderate to weak direct antifungal activity, so these compounds offer a dual mode of action with both direct inhibitory effect again *C. lindemuthianum* and the capacity of enhance the phytoalexin content, and consequently the plant resistance. 
